# HIV-1 Drug Resistance in Children and Implications for Pediatric Treatment Strategies: A Systematic Review and Meta-analysis

**DOI:** 10.1093/ofid/ofaf378

**Published:** 2025-06-26

**Authors:** Joseph Fokam, Aude Christelle Ka’e, Bouba Yagai, Maria Mercedes Santoro, Judith Kose Otieno, Natella Rakhmanina, Collins Ambe Chenwi, Alex Durand Nka, Ezechiel Ngoufack Jagni Semengue, Davy-Hyacinthe Gouissi, Willy Leroi Pabo Togna, Nelly Kamgaing, Tetang Suzie, Desire Takou, Georges Teto, Tatiana Tekoh, Jeremiah Efakika Gabisa, Audrey Nayang Mundo, Lum Forgwei, Naomi-Karell Etame, Aurelie Minelle Kengni Ngueko, Michel Carlos Tommo Tchouaket, Boris Tchounga, Patrice Tchendjou, Joelle Nounouce Bouba Pamen, Rogers Ajeh Awoh, Gregory-Edie Halle-Ekane, Giulia Cappelli, Alexis Ndjolo, Francesca Ceccherini-Silberstein, Vittorio Colizzi, Jean Kaseya, Nicaise Ndembi, Carlo Federico Perno

**Affiliations:** Laboratory of Virology, Chantal Biya International Reference Centre for Research on HIV/AIDS Prevention and Management (CIRCB), Yaoundé, Cameroon; Central Technical Group, National AIDS Control Committee, Yaoundé, Cameroon; National HIV Drug Resistance Working Group, Ministry of Public Health, Yaoundé, Cameroon; Faculty of Health Sciences, University of Buea, Buea, Cameroon; Laboratory of Virology, Chantal Biya International Reference Centre for Research on HIV/AIDS Prevention and Management (CIRCB), Yaoundé, Cameroon; Laboratory of Virology, Chantal Biya International Reference Centre for Research on HIV/AIDS Prevention and Management (CIRCB), Yaoundé, Cameroon; Faculty of Medicine and Surgery, UniCamillus-Saint Camillus International University of Health Sciences, Rome, Italy; Department of Experimental Medicine, University of Rome Tor Vergata, Rome, Italy; Africa Centres for Disease Prevention and Control, Addis Ababa, Ethiopia; Elizabeth Glaser Pediatric AIDS Foundation, Washington, DC, USA; Children's National Hospital, The George Washington University, Washington, DC, USA; Laboratory of Virology, Chantal Biya International Reference Centre for Research on HIV/AIDS Prevention and Management (CIRCB), Yaoundé, Cameroon; Department of Experimental Medicine, University of Rome Tor Vergata, Rome, Italy; Laboratory of Virology, Chantal Biya International Reference Centre for Research on HIV/AIDS Prevention and Management (CIRCB), Yaoundé, Cameroon; Laboratory of Virology, Chantal Biya International Reference Centre for Research on HIV/AIDS Prevention and Management (CIRCB), Yaoundé, Cameroon; Laboratory of Virology, Chantal Biya International Reference Centre for Research on HIV/AIDS Prevention and Management (CIRCB), Yaoundé, Cameroon; Laboratory of Virology, Chantal Biya International Reference Centre for Research on HIV/AIDS Prevention and Management (CIRCB), Yaoundé, Cameroon; Faculty of Health Sciences, University of Buea, Buea, Cameroon; Laboratory of Virology, Chantal Biya International Reference Centre for Research on HIV/AIDS Prevention and Management (CIRCB), Yaoundé, Cameroon; Department of Pediatrics, Essos Hospital Centre, Yaoundé, Cameroon; Laboratory of Virology, Chantal Biya International Reference Centre for Research on HIV/AIDS Prevention and Management (CIRCB), Yaoundé, Cameroon; Laboratory of Virology, Chantal Biya International Reference Centre for Research on HIV/AIDS Prevention and Management (CIRCB), Yaoundé, Cameroon; Laboratory of Virology, Chantal Biya International Reference Centre for Research on HIV/AIDS Prevention and Management (CIRCB), Yaoundé, Cameroon; Faculty of Health Sciences, University of Buea, Buea, Cameroon; Laboratory of Virology, Chantal Biya International Reference Centre for Research on HIV/AIDS Prevention and Management (CIRCB), Yaoundé, Cameroon; Laboratory of Virology, Chantal Biya International Reference Centre for Research on HIV/AIDS Prevention and Management (CIRCB), Yaoundé, Cameroon; Laboratory of Virology, Chantal Biya International Reference Centre for Research on HIV/AIDS Prevention and Management (CIRCB), Yaoundé, Cameroon; Laboratory of Virology, Chantal Biya International Reference Centre for Research on HIV/AIDS Prevention and Management (CIRCB), Yaoundé, Cameroon; Laboratory of Virology, Chantal Biya International Reference Centre for Research on HIV/AIDS Prevention and Management (CIRCB), Yaoundé, Cameroon; Department of Experimental Medicine, University of Rome Tor Vergata, Rome, Italy; Laboratory of Virology, Chantal Biya International Reference Centre for Research on HIV/AIDS Prevention and Management (CIRCB), Yaoundé, Cameroon; Elizabeth Glaser Pediatric AIDS Foundation, Country Office, Douala, Cameroon; Elizabeth Glaser Pediatric AIDS Foundation, Country Office, Douala, Cameroon; Faculty of Medicine and Biomedical Sciences, University of Yaoundé I, Yaoundé, Cameroon; Department of Financial Resources and Heritage, Ministry of Public Health, Yaoundé, Cameroon; Faculty of Health Sciences, University of Buea, Buea, Cameroon; HIV, Tuberculosis and Malaria Global Funds and Partners' Subvention Coordination Unit, Ministry of Public Health, Yaoundé, Cameroon; Faculty of Health Sciences, University of Buea, Buea, Cameroon; Laboratory of Virology, Chantal Biya International Reference Centre for Research on HIV/AIDS Prevention and Management (CIRCB), Yaoundé, Cameroon; National Research Council, Rome, Italy; Institute for Biological Systems, CNR, Rome, Italy; Laboratory of Virology, Chantal Biya International Reference Centre for Research on HIV/AIDS Prevention and Management (CIRCB), Yaoundé, Cameroon; Faculty of Medicine and Biomedical Sciences, University of Yaoundé I, Yaoundé, Cameroon; Department of Experimental Medicine, University of Rome Tor Vergata, Rome, Italy; Laboratory of Virology, Chantal Biya International Reference Centre for Research on HIV/AIDS Prevention and Management (CIRCB), Yaoundé, Cameroon; Department of Experimental Medicine, University of Rome Tor Vergata, Rome, Italy; Africa Centres for Disease Prevention and Control, Addis Ababa, Ethiopia; Africa Centres for Disease Prevention and Control, Addis Ababa, Ethiopia; Institute of Human Virology, University of Baltimore, Baltimore, Maryland, USA; Laboratory of Virology, Chantal Biya International Reference Centre for Research on HIV/AIDS Prevention and Management (CIRCB), Yaoundé, Cameroon; Bambino Gesu Pediatric Hospital, IRCCS, Rome, Italy

**Keywords:** ART, children, systematic review, “acquired drug resistance”, “pretreatment drug resistance”

## Abstract

**Introduction:**

Failure in the prevention of mother-to-child HIV transmission (PMTCT) and pediatric treatment challenges led to pretreatment drug resistance (PDR) and acquired drug resistance (ADR) in children with HIV (CWHIV).

**Method:**

Interventional and observational data published between 2010 and 2024 on PDR and ADR in CWHIV were included and analyzed by random effects models.

**Results:**

Overall, 72 studies encompassing 9973 children were included. The prevalence (95% CI) of PDR was 32.48% (26.08–39.21), and high among those who failed PMTCT prophylaxis (43.23% [32.94–53.82]) versus those without PMTCT-intervention (*P* < .01) and driven by nonnucleoside reverse transcriptase inhibitors (NNRTI) mutations (28.38% [18.74–39.08]; *P* = .013). The prevalence of ADR was 61.43% (49.82–72.45), driven by NNRTI-mutations (65.17% [53.95–75.63]; *P* < .001). INSTI-ADR was low (5.53% [2.49–9.53]) but emerging.

**Conclusion:**

There are high burdens of PDR and ADR among CWHIV, suggesting the need to phase out pediatric NNRTIs used for either PMTCT or treatment. Emerging INSTI resistance among CWHIV highlights the relevance of drug-resistance surveillance strategies.

**Prospero registration No:**

CRD42023470034.

The risk of perinatal HIV infection without any intervention varies from 15% to 45%, with about one-fourth of exposed newborns acquiring the virus during childbirth and one-fifth during pregnancy and breastfeeding [1]. This risk of vertical transmission has reduced substantially over time with the successful implementation of HIV prevention of mother-to-child transmission (PMTCT) through screening and management of both mothers and infants [2, 3]. In the current era, successful PMTCT programs are designed to achieve elimination targets by reducing HIV vertical transmission to less than 2% at 6–8 weeks and less than 5% after breastfeeding cessation throughout the PMTCT cascade care in low- and middle-income countries (LMICs) [4]. Of note, PMTCT has significantly contributed to the concept of treatment as prevention, owing to the impact of antiretrovirals in preventing HIV transmission to exposed children [5]. However, despite efforts to scale up PMTCT interventions globally, the burden of pediatric HIV infection is still of concern among exposed infants who have experienced PMTCT failure or without any PMTCT interventions. Suboptimal exposure to antiretroviral (ARV)-based prophylaxis during PMTCT increases the risk of pretreatment drug resistance (PDR) especially in LMICs like sub-Saharan Africa (SSA) where low genetic barrier drugs are still used. In SSA, about 159 000 children are newly infected with HIV every year and most of those identified are initiated on antiretroviral therapy (ART) following the test and treat strategy recommended by the World Health Organization (WHO) since 2016 [5–7].

Despite the pediatric ART rollout, children with HIV (CWHIV) are faced with limited treatment options and coverage, with only 57% of children aged 0–14 years accessing treatment in 2022 [8]. In July 2019, the WHO revised its recommendations regarding first- and second-line ARTs, following new evidence on the effectiveness and drug safety of preferred and alternative ART regimens [9]. More importantly, UNAIDS set a global 95-95-95 target aimed at ensuring that 95% of people with HIV know their status, 95% of those who know their status are receiving ART, and 95% of those receiving ART have achieved viral suppression by 2025 in all subpopulations, geographical settings and age groups including children and adolescents living with HIV [10]. By the end of 2023, these targets were at 86%, 89%, and 93% respectively, indicating hopes in achieving HIV elimination and sustained epidemic control beyond 2030 [10].

Achieving HIV elimination requires equitable access to, and effective use of efficacious and better tolerated ARVs [11, 12]. These lifesaving treatments have normalized the life expectancy of CWHIV, allowing them to grow toward adolescence and adulthood while maintaining lifetime ART [13]. This is particularly true following the rollout of dolutegravir (DTG), with rates of viral suppression reaching more than 90% of the general population receiving ART [14]. Nevertheless, viral suppression is not uniform across diverse regions and populations [15]. Besides the benefits of ART, the lifelong therapeutic exposure also raises concerns regarding toxicities, drug interactions, suboptimal adherence to ART (more frequent in pediatric populations, chiefly driven by nondisclosure and orphan hood), and the emergence of HIV drug resistance (HIVDR) [16]. Considering programmatic challenges in LMICs, inappropriate dispensing practices, recurrent drug stock-outs, poor retention in care, limited therapeutic options, socioeconomic disparities, and limited access to specialized medical care or reference therapeutic monitoring (viral load, CD4, genotypic resistance testing, adherence biomarkers, and drug safety assessment) in LMICs, the emergence of HIVDR is becoming even more concerning and outweighs gains at global level toward pandemic control [17, 18].

Suboptimal drug levels in plasma drive by HIVDR, inadequate dosing guidelines for specific ARVs and incomplete compliance, which are all more alarming in pediatric populations. For instance, previous studies have reported higher rates of HIVDR in children as compared to adults living with HIV [19, 20]. Both PDR and acquired drug resistance (ADR) represent major threats in children [21–26], based on the clinical significance of PDR on response to first-line ART. At the same time, ADR is known to compromise ART efficacy, which likely leads to viral rebound, HIV-associated morbidity/mortality, and increased risk of HIV transmission at population level [27, 28]. Hence, the added value of HIVDR genotyping cannot be overemphasized, and clinical HIVDR testing has become a standard of care in managing HIV infection, especially for CWHIV who need lifesaving treatments to safeguard their life expectancy.

With the goal to contribute to shaping treatment guidelines and monitoring strategies in pediatric populations at the global level, the present study aims to assess the global pooled prevalence of PDR and ADR as well as associated factors in CWHIV over the past decade.

## METHODS

### Design and Registration

This systematic review and meta-analysis was performed following the guidelines of Preferred Reporting Items for Systematic Review and Meta-Analyses [29] and was registered in the Prospective Register of Systematic Reviews (CRD42023470034).

### Design and Setting of the Study

*Inclusion criteria*

1. **Type of studies:** Randomized and nonrandomized trials, cohort and cross-sectional studies assessing data on HIVDR in CWHIV were included.
2. **Type of participants**: We considered studies conducted among CWHIV, aged between 0 and 15 years and as defined by WHO (www.who.int/news-room/fact-sheets/detail/hiv-aids), from all geographical locations worldwide.
3. **Intervention:** This entailed the implementation of PMTCT interventions among ART-naïve children. Regarding children receiving ARV treatment, intervention entails exposure to ART, and regimens were further stratified according to drug classes.
4. **Comparator:** Comparators were PMTCT exposure (with PMTCT exposure vs no PMTCT exposure), ART classes, and geographical locations (SSA vs other regions) [30].
5. **Types of outcomes:** Primary outcomes were the pooled prevalence of HIV-1 PDR and ADR in CWHIV. Secondary outcomes were (a) the identification of factors associated with PDR or ADR and (b) the implications for pediatric treatment strategies.
6. **Report characteristics:** We included studies published in English or French from 2010 to early 2024 or presented at IAS and the international workshops on HIV drug resistance and treatment strategies.

*Exclusion criteria*

Publications such as case reports, letters, comments, reviews, systematic reviews, and meta-analysis and editorials studies were excluded.

### Search Strategy

A systematic search was performed using PubMed/MEDLINE, Google Scholar, ScienceDirect, African journals online, and gray literature databases (conference abstracts) using the keywords: HIV-1, “drug resistance,” “acquired drug resistance,” “pretreatment drug resistance,” infants, children linked by the following Boolean operators: “OR” and “AND” (Supplementary File 1 shows the detailed search strategy for PubMed). A filter was performed by year (2010–2024). Additional studies were screened manually from the references of included studies.

### Selection of Studies for Inclusion in the Review

Records from the various sources were combined in an excel spreadsheet. Duplicates were identified and removed. Relevant studies were selected by 2 study authors (A.C.K. and A.D.N.) after an independent examination of the titles and abstracts of the eligible studies. Before data extraction, discordant ideas between investigators regarding the selection of the studies were resolved by discussion, consensus, or intervention of a third person (B.Y. or E.N.J.S.) when necessary.

### Data Extraction and Management

Four study authors (A.C.K., A.D.N., J.E.G., and T.T.) used a Google form questionnaire to extract and verify data from the included studies. The extracted data were: the name of the first author, the year of publication, the study design, the inclusion criteria, sampling method, sampling period, age, gender, sample size, sample type, the rate of HIVDR, the rate of HIVDR by classes, HIV-1 subtypes, PMTCT exposure (no PMTCT exposure vs PMTCT exposure), ART (drug class, ART regimen), WHO clinical staging (I, II, III, IV), and geographical location (country/continent) whenever available. Disagreements observed by different data extractors during data extraction were resolved by discussion and/or consensus.

### Data Analysis

*I* ^2^ and H statistics were used to estimate the heterogeneity among studies [31]. The *I*^2^ value indicates the degree of heterogeneity, with values of 0%, 18%, 45%, and 75% designating no, low, moderate, and high heterogeneity [32], respectively. Regarding the H statistics, lack of evidence on heterogeneity among studies was designated by obtaining an H value close to 1. Otherwise, these values were inversely proportional with the degree of heterogeneity. The pooled prevalence of PDR and ADR and 95% confidence intervals (95% CI) were estimated by random effect models [33]. Random effects meta-regression was used for subgroup analysis. PDR prevalence was categorized as low (<5%), moderate (5%–15%), or high (>15%) per WHO guidelines [34]. The R version 3.6.0 software (packages “meta,” “metafor,” and “ggplot 2”) was used to perform all meta-analysis, through the R studio interface [35, 36]. The dynamic curves of HIVDR were performed according to sampling years and trend proportion test was used to evaluate the significance of the evolution overtime.

### Risk of Bias Assessment

The quality of each study was independently assessed by 3 study authors (A.C.K., A.D.N., and J.E.G.) using a dedicated scale for prevalence studies that is based on 10 components divided into 2 groups: internal and external validity of the study (Supplementary File 2) [37]. A score of 0 or 1 was assigned to each question in the assessment tool for a total score of 10 per study. The scores of 0–3, 4–6, and 7–10 represented a high, moderate, and low risk of bias, respectively. For nonrandomized studies, the risk of bias was evaluated using ROBINS-1 [38], whereas ROBIS [RoB 2.0] was used for randomized controlled trials [39] (Supplementary File 3). Importantly, divergence in risk of bias assessment among the review authors was solved through discussion and consensus or by arbitration of a third review author.

## RESULTS

### Population Characteristics

**Figure 1.**
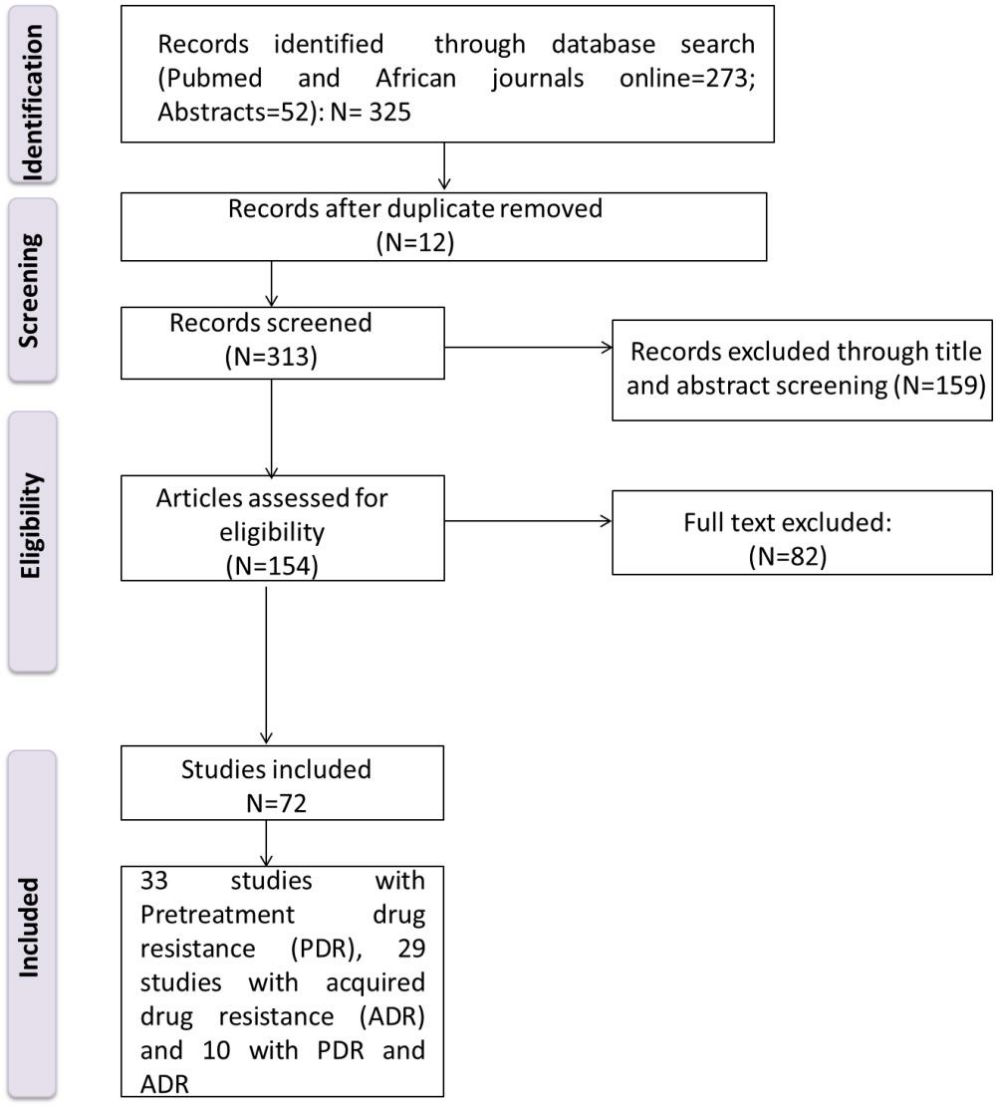
Flowchart of the study selection process.

A total of 325 studies were identified through the electronic search strategy. Duplicates (n = 12), irrelevant studies based on titles, and abstracts were removed (n = 159); 152 studies were assessed for full text eligibility and 72 studies finally met all inclusion criteria. Figure 1 shows the study selection process.

Overall, studies in this review encompassed 9973 children aged from 0 to 15 years with 52.5% being male and 47.5% female. Most (38.46%) of studies were cross-sectional, followed by clinical trials (21.7%), cohort studies (17.9%), surveys (2.56%), surveillance (1.28%), and chart review (1.28%). About 16.7% of studies included were without study design reported. Characteristics of the included studies are available on Table 1.

**Table 1.** Characteristics of Included Studies

Number	Author and Year of Publication	Study Design	Number of Sites	Country	Risk of Bias Assessment
1	Moraka et al, 2021 [40]	Nonrandomized clinical trial	Multicenter	Botswana	Low risk of bias
2	Vavro et al, 2022 [41]	Nonrandomized clinical trial	Multicenter	Botswana, Brazil, Kenya, South Africa, Tanzania, Thailand, USA, Zimbabwe	Low risk of bias
3	Violari et al, 2015 [42]	Nonrandomized clinical trial	Multicenter	Argentina, Brazil, India, South Africa	Low risk of bias
4	Fogel et al, 2013 [43]	Randomized clinical trial	Not/reported/unclear	Uganda	Low risk of bias
5	Harrison et al, 2015 [44]	Randomized clinical trial	Multicenter	Europe and North and South America	Low risk of bias
6	Nacro et al, 2011 [45]	Nonrandomized clinical trial	Multicenter	Burkina Faso	Low risk of bias
7	Beck et al, 2012 [46]	Randomized clinical trial	Not reported	USA	Low risk of bias
8	Inzaule et al, 2016 [47]	Nonrandomized clinical trial	Not reported	Kenya	Moderate risk of bias
9	Boyce et al, 2023 [48]	Randomized clinical trial	Multicenter	Botswana, Kenya, South Africa, Tanzania, Thailand, Uganda, USA, Zimbabwe	Low risk of bias
10	Tambuyzer et al, 2016 [49]	Nonrandomized clinical trial	Multicenter	Belgium, France, Brazil, Puerto Rico, USA, Portugal, United Kingdom, Thailand, Romania, Italy, Netherlands, Canada, South Africa	Low risk of bias
11	Fokam et al, 2011 [50]	Cross-sectional study	Monocenter	Cameroon	Low risk of bias
12	Musiime et al, 2016 [51]	Randomized clinical trial	Multicenter	Uganda, Zimbabwe	Low risk of bias
13	Barro et al, 2011 [52]	Nonrandomized clinical trial	Monocenter	Burkina Faso	Low risk of bias
14	Huibers et al, 2019 [53]	Cohort	Monocenter	Uganda	Low risk of bias
15	Kuhn et al, 2012 [54]	Randomized clinical trial	Monocenter	South Africa	Low risk of bias
16	Butler et al, 2016 [55]	Randomized clinical trial	Multicenter	Belgium, Denmark, Germany, Ireland, Spain, Thailand, Uganda, United Kingdom, Argentina, Ukraine and the USA	Low risk of bias
17	Dahourou et al, 2017 [56]	Randomized clinical trial	Multicenter	Burkina Faso and Ivory Coast	Low risk of bias
18	Olusola et al, 2021 [57]	Cross-sectional study	Multicenter	Nigeria	Low risk of bias
19	Parham et al, 2011 [58]	Cross-sectional study	Multicenter	Honduras and Belize	Low risk of bias
20	Ferreira et al, 2010 [59]	Cross-sectional study	Monocenter	Brazil	Moderate risk of bias
21	Charpentier et al, 2011 [60]	Cross-sectional study	Monocenter	Central African Republic	Moderate risk of bias
22	Mulder et al, 2011 [61]	Cohort	Multicenter	Spain	Low risk of bias
23	Almeida et al, 2012 [25]	Cohort	Monocenter	Brazil	Low risk of bias
24	Mulder et al, 2012 [62]	Not reported/unclear	Multicenter	Spain	Moderate risk of bias
25	Neogi et al, 2012 [63]	Cross-sectional study	Multicenter	India	Low risk of bias
26	Shao et al, 2014 [64]	Cross-sectional study	Multicenter	Tanzania	Low risk of bias
					
					
27	Andrade and Sabid, 2017 [65]	Cohort	Monocenter	Brazil	Low risk of bias
28	Kityo et al, 2017 [21]	Cohort	Multicenter	Uganda	Low risk of bias
29	Jordan et al, 2017 [66]	Surveillance	Multicenter	Uganda, Zimbabwe, Swaziland, South Africa, Mozambique	Low risk of bias
30	Kanthula et al, 2017 [67]	Cohort	Monocenter	South Africa	Low risk of bias
31	Poppe et al, 2017 [68]	Not reported/unclear	Monocenter	Zambia	Moderate risk of bias
32	Yeganeh et al, 2017 [69]	Not reported/unclear	Multicenter	South Africa, Brazil and Argentina	Low risk of bias
33	Aulicino et al, 2018 [70]	Not reported/unclear	Multicenter	Argentina	Moderate risk of bias
34	Frange et al, 2018 [71]	Not reported/unclear	Multicenter	France	Moderate risk of bias
35	Hunt et al, 2019 [72]	Cross-sectional	Multicenter	South Africa	Low risk of bias
					
36	Tadesse et al, 2019 [24]	Cross-sectional study	Multicenter	Ethiopia	Low risk of bias
37	Bennett et al, 2020 [73]	Not reported/unclear	Monocenter	Zambia	Moderate risk of bias
38	Dambaya et al, 2020 [22]	Cross-sectional study	Multicenter	Cameroon	Low risk of bias
39	Boerma et al, 2016 [74]	Cohort	Monocenter	Nigeria	Low risk of bias
40	Dow et al, 2017 [75]	Cross-sectional study	Multicenter	Tanzania	Low risk of bias
41	Namayanja et al, 2023 [76]	Cross-sectional survey	Multicenter	Uganda	Low risk of bias
42	Boyce et al, 2023 [48]	Cohort	Multicenter	Botswana, Kenya, South Africa, Tanzania, Thailand, Uganda, USA, Zimbabwe	Low risk of bias
43	Hackett et al, 2023 [77]	Cohort	Monocenter	Uganda	Low risk of bias
44	Bratholm et al, 2012 [78]	Cross-sectional study	Monocenter	Tanzania	Moderate risk of bias
45	Mohamad et al, 2012 [79]	Cross-sectional study	Monocenter	Malaysia	Moderate risk of bias
46	Tolle et al, 2012 [80]	Cross-sectional study	Monocenter	Botswana	Moderate risk of bias
47	Gomila et al, 2013 [81]	Cross-sectional study	Monocenter	Botswana	Moderate risk of bias
48	Salou et al, 2016 [82]	Cross-sectional study	Nationally representative	Togo	Low risk of bias
49	Mossoro-Kpinde et al, 2017 [83]	Cross-sectional study	Monocenter	Central African Republic	Moderate risk of bias
50	Muri et al, 2017 [84]	Cohort	Monocenter	Tanzania	Low risk of bias
51	Fofana et al, 2018 [85]	Cross-sectional study	Monocenter	Benin	Low risk of bias
52	Tadesse et al, 2018 [86]	Cross-sectional study	Monocenter	South Africa	Moderate risk of bias
53	Vaz et al, 2018 [87]	Cross-sectional study	Multicenter	Mozambique	Low risk of bias
54	Cissé et al, 2019 [88]	Cross-sectional study	Multicenter	Senegal	Low risk of bias
55	Bouassa et al, 2019 [89]	Cohort	Monocenter	Central African Republic	Low risk of bias
56	Soumah et al, 2019 [90]	Not reported/unclear	Multicenter	France/sub-Saharan Africa	Moderate risk of bias
57	Hackett et al, 2021 [91]	Cohort	Multicenter	South Africa	Low risk of bias
58	Tagnouokam-Ngoupo et al, 2021 [92]	Cohort	Multicenter	Cameroon	Low risk of bias
59	Soeria-Atmadja et al, 2019 [93]	Cohort	NR	Uganda	Moderate risk of bias
60	Chaplin et al, 2018 [94]	Cross-sectional study	Multicenter	Nigeria	Low risk of bias
61	Rozenszajn et al, 2022 [95]	NR	NR	Argentina	High risk of bias
62	Jordan et al, 2022 [96]	Survey	Multicenter	Namibia	Low risk of bias
63	Ebonyi et al, 2024 [97]	NR	NR	Nigeria	High risk of bias
64	Karunaianantham et al, 2022 [26]	NR	NR	India	Moderate risk of bias
65	Kamori et al, 2023 [98]	Cross-sectional study	Multicenter	Tanzania	Low risk of bias
66	Fisher et al, 2015 [99]	Cross-sectional study	NR	South Africa	High risk of bias
67	Holguin et al, 2011 [100]	Cohort	Multicenter	Honduras, El Salvador	Low risk of bias
68	Antunes et al, 2015 [101]	Cross-sectional	Multicenter	Mozambique	Low risk of bias
69	Patel et al, 2023 [102]	Randomized clinical trial	Multicenter	Kenya	Low risk of bias
70	Kébé et al, 2014 [103]	Cross-sectional study	Monocenter	Senegal	Moderate risk of bias
71	Inzaule et al, 2018 [104]	Cross-sectional study	Multicenter	Nigeria	Low risk of bias
72	Louis et al, 2019 [105]	Not reported/unclear	Multicenter	Haiti	Moderate risk of bias

### Rates of Pretreatment and Acquired Drug Resistance

**Figure 2.**
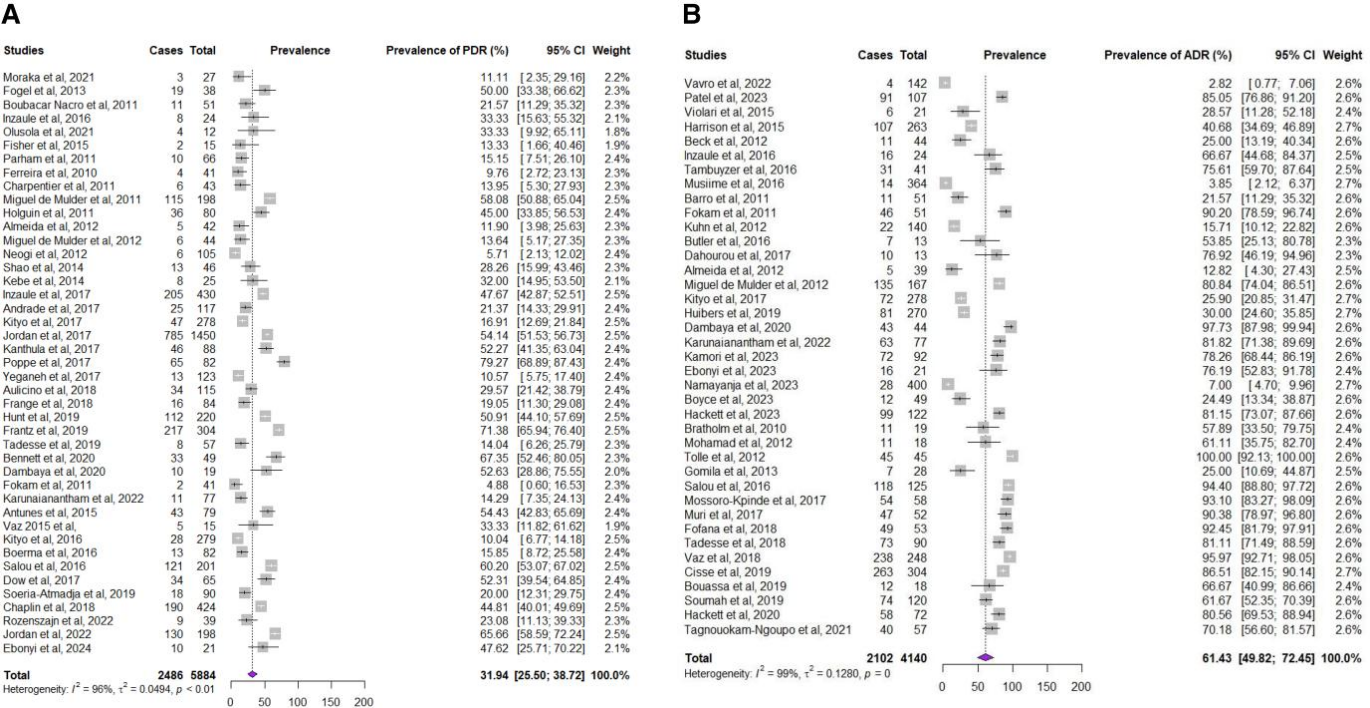
Pooled prevalence of pretreatment (*A*) and acquired drug resistance (*B*).

**Figure 3.**
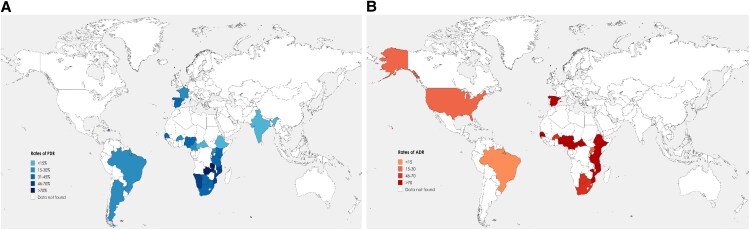
Geographical distribution of PDR (*A*) and ADR (*B*) prevalence between 2010 and early 2024. The cartography was performed considering studies included in the meta-analysis. ADR, acquired drug resistance; PDR, pretreatment drug resistance. Copyrighted: The figure was conceived and adapted by the authors of this review; the base layer of the map was downloaded from http://viewer.nationalmap.gov/viewer/.

Data on PDR [21–26, 40, 43, 45, 47, 50, 57–75, 93–97, 99–101, 103–106] were available for 5884 participants. Regarding PMTCT exposure, information was available for 3836 participants with 66.36% being exposed to PMTCT prophylaxis. Globally, the pooled prevalence [95% CI] of PDR was 31.94% [25.50–38.72]; Figure 2*A*. The prevalence among participants exposed to PMTCT prophylaxis was high, about 43.23% [32.94–53.82] compared to 19.40% [14.45–24.84] among those without any PMTCT exposure, *P* < .01. With respect to regional distribution, burden of pooled prevalence was high in SSA with a prevalence of 39.13% [31.36–47.17] as compared to other regions where the pooled prevalence was 22.56% [14.25–32.07]; *P* < .01. The distribution of PDR worldwide is shown in Figure 3*A*.

Data on ADR [21, 22, 25, 26, 41, 42, 44, 46–49, 51–56, 76, 78–92, 97, 98, 102] were available for 4140 individuals. The pooled prevalence of ADR was 61.43% [49.82–72.45] among children experiencing virological failure; Figure 2*B*. Regarding the situation of ADR according to countries, only 28/37 studies specified the country where the study was conducted. ADR was predominant in SSA compared to other geographical areas with 67.58% [53.24–80.48] versus 18.75% [8.34–31.87]; *P* < .01. The distribution of ADR worldwide is shown in Figure 3*B*.

### Patterns of Pretreatment Drug Resistance

According to drug classes, PDR was driven essentially by nonnucleoside reverse transcriptase inhibitors (NNRTI) (OR [95% CI]: 2.46 [2.12–7.86]; *P* = .013) with a prevalence of 28.38% [18.74–39.08], followed by nucleoside reverse transcriptase inhibitors (NRTI) with a prevalence of 12.06% [5.97–19.73], protease inhibitor (PI) with 5.51% [1.75–10.83] and integrase strand transfer inhibitor (INSTI) with 1.29% [0.0–7.37]; *P* < .01. NRTI resistance-associated mutations such as M184V, K65R, Y115F, Y115F, L74V, thymidine analogue mutations (TAMs) M41L, D67N, K70R, L210W, T215 revertants, and K219Q were reported [21, 25, 47, 58, 59, 62, 107]. NNRTI resistance-associated mutations mostly reported were K101E, K103S, E138A, E138K, E138Q, H221Y, M230L, K103N, V106A, V106M, V108I, Y181C, Y181I, Y188C, Y188H, Y188L, G190A, G190S, and P225H [21, 25, 40, 47, 58, 59, 62, 107]. PI resistance-associated mutations reported were D30N, K20R, M36I, I62V, L63I, L10I, I13V, M36I, M46I, L63A, L10V, L63S, V77I, L63H, I93L, L63P, I93L, L63T, I64V, L63R, L63E, D60E, I62V, L63AITV, and L10V [40, 58, 59, 82, 100]. INSTI resistance-associated mutations reported were G140R, E138K, and R263K [40, 48] and accessory mutations described were G163R, G140E, and G140K [40]. Additionally, E157Q, which is known to affect the susceptibility to raltegravir/elvitegravir rather than to dolutegravir, was also present [71]. PDR was associated with high pre-ART viral load and WHO clinical stage II (compared to WHO clinical stage I) [21].

### Patterns of Acquired Drug Resistance

As concerns the distribution of ADR by drug classes, NNRTI-resistance was the most predominant (OR [95% CI]: 3.84 [3.36–4.38]; *P* < .001) with a pooled prevalence of 65.17% [53.95–75.63] for NNRTI resistance, 53.24% [42.13–64.20] for NRTI resistance (indicating a remarkable increase of NRTI resistance in ADR compared to PDR), 8.37% [3.02–15.64] for PI resistance and 5.53% [2.49–9.53] for INSTI resistance; *P* < .01. NRTI resistance-associated mutations such as M184V, K65R, Y115F, Y115F, L74V, F77L, R211K, R211S, E44K, T69ND, L210G, G333EG, TAMs M41L, D67N, K70R, L210W, T215 revertants, and K219Q/E were reported [41, 42, 83, 86, 88, 91, 92, 107]. NNRTI resistance mutations reported were A98G, K101E, K103N, Y181C, E138A, E138Q, V106M, H221Y, G190A, G190GA, V179D, L100I, L100LI, F227L, Y188L, Y188FL, and P225PH [21, 41, 54, 83, 86, 88, 91, 92, 107] and PI resistance mutations were F53L, I47V, M46I, V77I, D60E, I54V, V82A/F, V11I, I62V, I15V, I63P, G16E, L10I/M/V, I13V, K20I/R, L89I/M/V, H69 K/R/Q/Y, and M36I/L [41, 42, 83, 86, 91, 92, 107]. Of note, dolutegravir-related mutations were found, such as E138K, G118R, G140A, G148K, R263K, and T66A [41, 76, 77, 98, 108]. Secondary integrase substitution E157Q and L74I were also reported [41].

### Dynamic of Pretreatment and Acquired Drug Resistance

**Figure 4.**
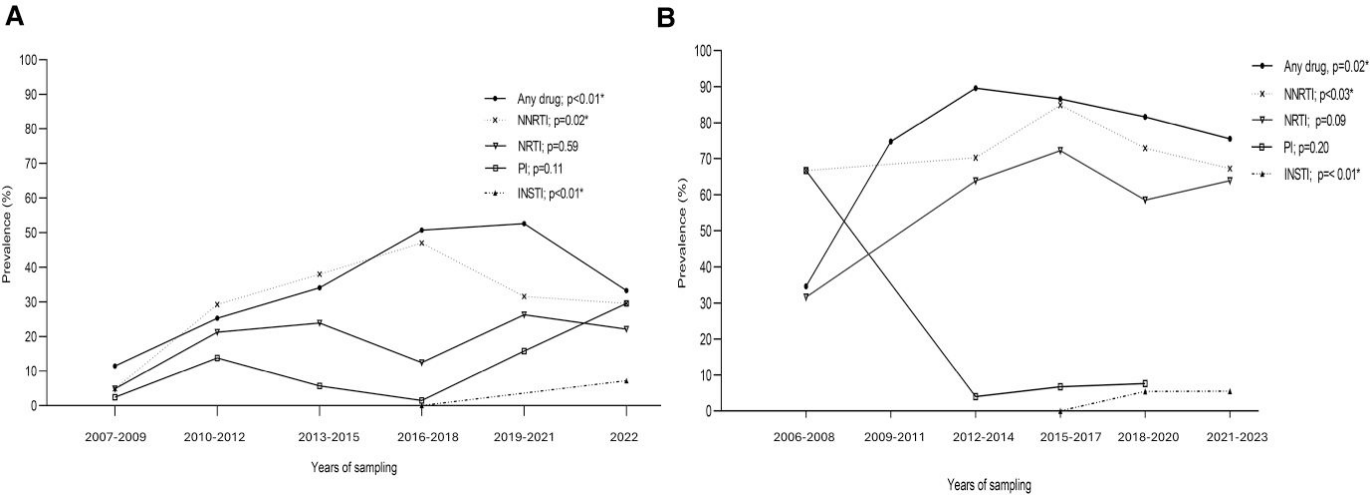
Trends of pretreatment drug resistance (PDR) and acquired drug resistance (ADR) by median year of sampling. Proportion trend test was used to evaluate the trend of HIVDR (*P* < .01 for each model). Each circle represents a study, and the diameter of the circle is proportional to the sample size of each study. The line fitted was plotted using regression analysis. (*A*) PDR and (*B*) ADR. HIVDR, HIV drug resistance.

As shown in Figure 4*A*, significant increase of PDR was observed between 2007 and 2018 (from 11.5% to 50.7%); the burden remains relatively the same between 2018 and 2021, when the DTG was rolled out. Nonetheless, a decrease was noted between 2021 and 2022 (from 52.6% to 33.3%), the period when DTG was fully expanded. However, during this same period, increase rates of PI and INSTI-PDR were observed (from 15.8% in 2019–2021 to 29.6% in 2022 for PI and from 0 in 2016 to 7.2% for INSTI in 2022). It is worth noting that between 2019 and 2022, NNRTI-PDR rate has not changed (∼30%). Concerning ADR, the rate has increased from 34.6% from 2006 to 86.59% in 2014 followed by a plateau phase from 2014 to 2023. A high rate (66.7%) of PI-ADR was observed between 2006 and 2008 in participants who failed first and second line of treatment. However, a decrease was noted from 66.7% in 2006 to 4% in 2014 followed by a plateau phase from 2014 to 2020. Of note, INSTI-ADR is emerging from 0% in 2015 to 5.5% in 2023 (after the rollout of DTG in 2019) (Figure 4*B*).

## DISCUSSION

In the global landscape of pediatric HIVDR prevention and control, recent systematic review and meta-analysis on HIVDR among children focused only on PDR solely in LMICs [109] and in SSA [110]. Another review without meta-analysis from Sanchez et al. evaluated HIVDR in pediatric populations but was published almost 10 years ago. To the best of our knowledge, the present study therefore seems to be the first systematic review and meta-analysis including more recent studies to describe the profile of both PDR and ADR worldwide in CWHIV over the past decade. As concerns data found, this systematic review has shown a lack of information on HIVDR in many countries from different continents, even in SSA countries with high burden of pediatric HIV infection.

This study shows that the pooled prevalence of PDR is very high, more than 30% and was fitted in a trend of increasing levels in SSA as previously shown by Boerma et al. in 2017 who also reported an alarming rate of PDR [110]. In fact, the prevalence of PDR in SSA regions was high as compared to other regions and might be explained by the previously extended use of low genetic-barrier drugs such as nevirapine prophylaxis during PMTCT intervention among infants after childbirth or efavirenz-containing regimens used to treat pregnant and breastfeeding women [72, 75]. More importantly, the prevalence of PDR was almost 3 times higher among infants with PMTCT exposure in comparison to those with no PMTCT intervention. This high prevalence (>40%) of PDR after PMTCT exposure is explained previously by the use of low genetic barrier drugs as prophylaxis during PMTCT intervention or maternal subtherapeutic regimens during pregnancy or breastfeeding [110], as supported by the WHO report showing that approximately half of CWHIV harbor PDR among selected surveys [111]. Moreover, despite the rollout of DTG since 2019, the observed persistent rate of NNRTI-PDR might be explained by the viral fitness of virus harboring NNRTI mutations (including Y181C and K103N) on the one hand and the continuous use of NNRTI based-prophylaxis regimen in SSA countries on the other. At the population level, our findings indicate that 3 to 4 of 10 CWHIV harbor PDR, in accordance with WHO findings. Importantly, this study also underscores a high prevalence (>15%) of PDR among PMTCT unexposed children, highlighting the importance of following the WHO recommendations in monitoring HIVDR among newly diagnosed infants. In addition to phasing out nevirapine in pediatric ART regimens, public health PMTCT measures to overcome this challenge include: (1) increasing the coverage of maternal DTG-based regimens to favor viral suppression of pregnant and breastfeeding women; (2) introduce newer infant prophylactic ARVs (INSTIs such as raltegravir, capsid inhibitors such as lenacapavir, etc) to replace the nevirapine prophylaxis in the frame of PMTCT cascade care; and (3) implementing the use of triple ARV prophylaxis known to be safe at birth (preferentially with higher genetic barriers) especially in SSA where HIV vertical transmission and PDR are very concerning. The low rate of INSTI-PDR supports the WHO recommendation to use DTG-based regimens as prefer first-line ART in CWHIV.

Overall rates of ADR in this pediatric population were also high and driven by NNRTI resistance, with higher levels in SSA (>65%) as compared to other regions. As factors associated with the development of ADR might include regimen-related factors, patient factors, viral factors, and program-related factors, the phasing out of low genetic barrier (nevirapine and Efavirenz) should be accompanied by efforts to widen the use of pediatric DTG-containing regimens, strengthen the supply chain management to prevent drug stock outs, ensure timely treatment monitoring with viral load, and better position the use of other important regimens such as ritonavir-boosted darunavir (so far with rare availability) or anticipate with injectable ARVs such as CARLA (cabotegravir-rilpivirine long acting) and lenacapavir (capsid inhibitor), based on the absence or low prevalence of ADR (∼5%) to these newer drugs [112]. Regarding viral factors, resistance mutation patterns may differ across subtypes, replicative fitness varies by viral clades, and it is demonstrated that the risk to ADR after exposure to single-dose nevirapine is high in HIV-1 subtype D as compared to HIV-1 subtype A, as well as the risk of pathogenesis [113]. Moreover, another example is that natural polymorphisms in reverse transcriptase of subtype C increase the propensity of the virus to select K65R in comparison to subtype B, resulting to tenofovir loss of efficacy even before the use of this drug [114]. Indeed, SSA harbors wide HIV genetic diversity, which may influence treatment outcomes and possibly the selection of HIVDR. This raises the need to also establish personalized treatment for cases of multi-resistance or heavily treated children/adolescents. Finally, ongoing vertical transmission and the limited pediatric ART is a call for developing newer ARVs with high safety during pregnancy and infancy. Such efforts, coupled to adequate maternal adherence counseling, male partner involvement, will contribute to achieve the elimination of pediatric AIDS [111–113]. It is, therefore, pivotal to strengthen health systems in LMICs, set up national public health institutes, and develop a thorough sustainability roadmap and strategic plan to achieve and maintain optimal response for PMTCT and to pediatric HIV management after epidemic control beyond 2030.

## CONCLUSION

This systematic review and metanalysis underscore the high prevalence of both PDR and ADR, driven by NNRTI in CWHIV, especially in SSAcompared to other regions. This emphasizes the need for NNRTI-sparing regimens for both prophylaxis and treatment in pediatrics. Furthermore, high PDR rates among PMTCT-exposed and PMTCT-unexposed children underscore the need for PDR surveillance to select fully active pediatric first-line ART in LMICs; ADR surveillance to design optimal pediatric ART strategies for second- and third-line ART to mitigate the growing rates of resistance to pediatric DTG. Overall, the threat of HIVDR in children calls for newer ARV prophylactic and therapeutic strategies, personalized clinical management, while advocating for the development of newer pediatric ARVs to fast-track the elimination of AIDS in this vulnerable population.

## Supplementary Material

ofaf378_Supplementary_Data

## Supplementary Data

Supplementary materials are available at *Open Forum Infectious Diseases* online. Consisting of data provided by the authors to benefit the reader, the posted materials are not copyedited and are the sole responsibility of the authors, so questions or comments should be addressed to the corresponding author.
