# Supplementary material for: HIV-1 Drug Resistance in Children and Implications for Pediatric Treatment Strategies: A Systematic Review and Meta-analysis
Source: Open Forum Infect Dis. 2025 Jun 26;12(7):ofaf378. doi: 10.1093/ofid/ofaf378 (PMC12282363; doi:10.1093/ofid/ofaf378)
Supplement: ofaf378_Supplementary_Data [file ofaf378_supplementary_data.zip › Supplementary file 1.docx]

Supplementary file 1: Search strategy in Medline (Pubmed)

| **Search** | **Key words** |
| --- | --- |
| #1 | “HIV-1” OR “Human Immunodeficiency Virus” |
| #2 | “HIV drug resistance” OR “pretreatment drug resistance” OR PDR OR “Acquired drug resistance” OR ADR |
| #3 | Infants OR Children |
| #4 | #1 AND #2 AND #3 |
