# Supplementary material for: HIV-1 Drug Resistance in Children and Implications for Pediatric Treatment Strategies: A Systematic Review and Meta-analysis
Source: Open Forum Infect Dis. 2025 Jun 26;12(7):ofaf378. doi: 10.1093/ofid/ofaf378 (PMC12282363; doi:10.1093/ofid/ofaf378)
Supplement: ofaf378_Supplementary_Data [file ofaf378_supplementary_data.zip › Supplementary file 2.docx]

**Supplementary file 2**: Items for risk of bias assessment for prevalence studies

| **Hoy *et al*. tool** | **Yes (1)/No (0)** |
| --- | --- |
| **External validity** |  |
| 1. Was the study’s target population a close representation of the national population in relation to HIV prevalence? | **1** |
| 2. Was the sampling frame a true or close representation of the study population? | **1** |
| 3. Was some form of random selection used to select the sample, OR was a census undertaken? | **1** |
| 4. Was the likelihood of nonresponse bias minimal (> 70%)? | **1** |
| **Internal validity** | **1** |
| 5. Were data collected directly from the subjects (as opposed to a proxy)? | **1** |
| 6. Was an acceptable case definition used in the study? | **1** |
| 7. Was the study viral detection assay shown to have validity and reliability? | **1** |
| 8. Was the same mode type of sample collected for all subjects? | **1** |
| 9. Was the length of the length of the study period > 1 year? | **1** |
| 10. Were the numerator(s) and denominator(s) for the resistance prevalence appropriate? | **1** |
| Total score | **10** |
| **Interpretation of the risk of bias tool** • 7-10: Low risk of bias • 4-6: Moderate risk of bias • 0-3: High risk of bias |  |

Hoy D, Brooks P, Woolf A, Blyth F, March L, Bain C, et al. Assessing risk of bias in prevalence studies: modification of an existing tool and evidence of interrater agreement. J Clin Epidemiol. 2012; 65: 934–939. https://doi.org/10.1016/j.jclinepi.2011.11.014 PMID: 22742910
