# Supplementary material for: HIV-1 Drug Resistance in Children and Implications for Pediatric Treatment Strategies: A Systematic Review and Meta-analysis
Source: Open Forum Infect Dis. 2025 Jun 26;12(7):ofaf378. doi: 10.1093/ofid/ofaf378 (PMC12282363; doi:10.1093/ofid/ofaf378)
Supplement: ofaf378_Supplementary_Data [file ofaf378_supplementary_data.zip › Supplementary file 3.docx]

**Supplementary file 3**: Assessing the quality of evidence and the strength of recommendations of randomized and non-randomized studies.

| **Types of studies** | **Risks of bias** | **Interpretation** | **Quality of evidence** | **Strength of the recommendation** |
| --- | --- | --- | --- | --- |
| Randomized studies | Low risk of bias | Most information is from studies at low risk of bias. | High | Strong |
|  | Unclear risk of bias | Most information is from studies at low or unclear risk of bias. | Moderate | Moderate |
|  |  |  | Low |  |
| Non-randomized studies | High risk of bias | The proportion of information from studies at high risk of bias is sufficient to affect the interpretation of results. | Very Low | Weak |
